# Apigenin-7-*O*-*β*-D-(-6”-*p*-coumaroyl)-glucopyranoside treatment elicits a neuroprotective effect through GSK-3β phosphorylation-mediated Nrf2 activation

**DOI:** 10.18632/aging.104050

**Published:** 2020-11-18

**Authors:** Jingwen Wang, Shiquan Wang, Sisi Sun, Yunyang Lu, Kai Gao, Chao Guo, Ruili Li, Weiwei Li, Xian Zhao, Haifeng Tang, Aidong Wen, Min Cai, Wei Zhang

**Affiliations:** 1 Department of Pharmacy, Xijing Hospital, The Fourth Military Medical School, Xi'an 710032, Shaanxi, China; 2 Department of Anesthesiology and Perioperative Medicine, Xijing Hospital, The Fourth Military Medical School, Xi'an 710032, Shaanxi, China; 3 The Medical Department of the Emergence Centre of Xi'an 718900, Shaanxi, China; 4 Institute of Materia Medica, School of Pharmacy, The Fourth Military Medical School, Xi'an 710032, Shaanxi, China; 5 Department of Psychiatry, Xijing Hospital, The Fourth Military Medical University, Xi’an 710032, Shaanxi Province, China

**Keywords:** Apigenin-7-*O*-*β*-D-(-6”-*p*-coumaroyl)-glucopyranoside, ischemic stroke, glycogen synthase kinase-3β, NF-E2-related factor 2, oxidative stress

## Abstract

The current study was designed to seek the role of the glycogen synthase kinase-3β (GSK-β)-regulated NF-E2-related factor 2 (Nrf2) pathway in the antioxidant effect induced by Apigenin-7-O-β-D-(-6”-p-coumaroyl)-glucopyranoside (APG). Rat primary cultured cortical neurons were challenged by oxygen and glucose deprivation/reoxygenation (OGD/R) and then treated with APG. Cell viability, phosphorylation of GSK-β at Ser9 and nuclear expression of Nrf2 were measured. Male Sprague Dawley rats challenged by 2-h middle cerebral artery occlusion were treated with 50 mg/kg APG, and the neurological score, infarct volume, phosphorylation of GSK-3β and nuclear expression of Nrf2 were analyzed. The neuroprotective effect of APG and the expression levels of antioxidant enzymes and oxidative products were also examined in the presence and absence of Nrf2-siRNA and PI3K inhibitors. APG reduced the apoptotic proportion, attenuated LDH release and increased cell viability, and *in vivo,* APG improved neurological scores and reduced infarct volume. APG increased GSK-3β phosphorylation and Nrf2 nuclear translocation, while these effects were prevented by PI3K inhibitors or Nrf2-siRNA treatment in both OGD/R cell cultures and ischemic/reperfusion rats. These findings reveal that GSK-3β phosphorylation-mediated Nrf2 activation is involved in the neuroprotective effect of APG.

## INTRODUCTION

Accumulating evidence has determined that oxidative stress plays a crucial role in the pathogenesis of cerebral ischemic/reperfusion (I/R) injury [1]. Unfortunately, despite this importance in the pathophysiological process of I/R, exogenous antioxidant supplementation targeting oxidative stress failed to show a favorable outcome against stroke in clinical trials [2, 3]. Meanwhile, an improvement in the endogenous antioxidant system was found to elicit a proposed self-protective mechanism in the brain itself against cerebral I/R damage, which reveals that a therapeutic approach targeting the endogenous antioxidant system may be a potential avenue for treatment of ischemic stroke [4–7].

As the major determinant of redox homeostasis against cellular stress, NF-E2-related factor 2 (Nrf2) primarily functions as a transcription activator in the nucleus in partnership with small Mafs. Nrf2 has been found to active the expression of antioxidant-response and phase II detoxifying genes, which contain an enhancer sequence termed antioxidant response element (ARE) in their promoter regulatory regions [8, 9]. These genes encode a large number of cytoprotective proteins involved in antioxidant reactions, including the anti-inflammation response, neurogenesis, and mitochondrial genesis, among others [10–12]. Some neuroprotective therapies, including pharmacological treatments and traditional Chinese medicines, have been demonstrated to exert their protective benefits by enhancing the Nrf2-mediated endogenous antioxidant defense system [12–16]. However, whether this signaling pathway is involved in the ischemia tolerance produced by APG has not yet been clarified.

Nrf2 regulation is principally mediated by two different mechanisms. The first established mechanism is protein stability controlled by Kelch-like ECH-associated protein 1 (Keap1), which acts as a ubiquitin E3 ligase substrate adapter for a Cullin3/Rbx1-dependent E3 ubiquitination pathway [17, 18]. Under physiological conditions, Nrf2 is located in the cytoplasm and binds to Keap1, leading to Nrf2 degradation via 26 s proteasome-mediated ubiquitination [19, 20]. The other mechanism involves glycogen synthase kinase-3β (GSK-3β), which phosphorylates Nrf2 at a site for recognition of β-transducin repeat containing E3 ubiquitin protein ligase (β-TrCP) [21]. β-TrCP mediates cullin-1/Rbx1-regulated Nrf2 ubiquitination and degradation, and this is a major contributor to the pathogenesis of cerebral I/R [22–25]. Importantly, in addition to regulating physiological processes, GSK-3β is also involved in the cellular response to various damage insults, including the development of cerebral ischemia [26–28]. Phosphorylation of GSK-3β at Ser-9 represents the inhibited state of this protein, which enables cells to resistant various pathophysiological insults more effectively. However, dephosphorylation of GSK-3β reveals worse dampened cellular tolerance to such damage insults [24, 29, 30]. Additionally, recent studies have found that GSK-3β participates as a negative regulator of Nrf2 by influencing the distribution of Nrf2 in the cytoplasm and nucleus [22]. This underlying mechanism regulates Nrf2 in a Keap-1 independent manner and is a major determinant in the prognosis of cerebral I/R injury [31]. Because GSK-3β is inactivated through phosphorylation at Ser9 by Ser/Thr protein kinases, such as phosphatidylinositol 3-kinase (PI3K), GSK-3β phosphorylation could potentially promote Nrf2 activation [32, 33].

In a previous study, we reported the protective effect of apigenin-7-*O*-*β*-D-(-6”-*p*-coumaroyl)-glucopyranoside (APG), a glycoside subtype of apigenin extracted from the traditional Chinese herb *Clematis tangutica,* that exhibited a neuroprotective effect in both *in vitro* and *in vivo* cerebral ischemia/reperfusion models [34]. This study also indicated that the neuroprotective effect of APG was associated with activation of the endogenous antioxidant system. However, the mechanism underlying the antioxidant response produced by APG needs to be further explored. Therefore, in the present study, we sought to analyze the effect of APG on Nrf2 activation induced by oxygen and glucose deprivation and reperfusion (OGD/R) in primary cultured rat cortical neurons and in rats subjected to middle cerebral artery occlusion (MCAO). We also aimed to determine whether the Nrf2 nuclear translocation promoted by APG treatment was modulated by GSK-3β phosphorylation at Ser-9.

## RESULTS

### APG exerted a protective effect against OGD/R injury

**Figure 1:**
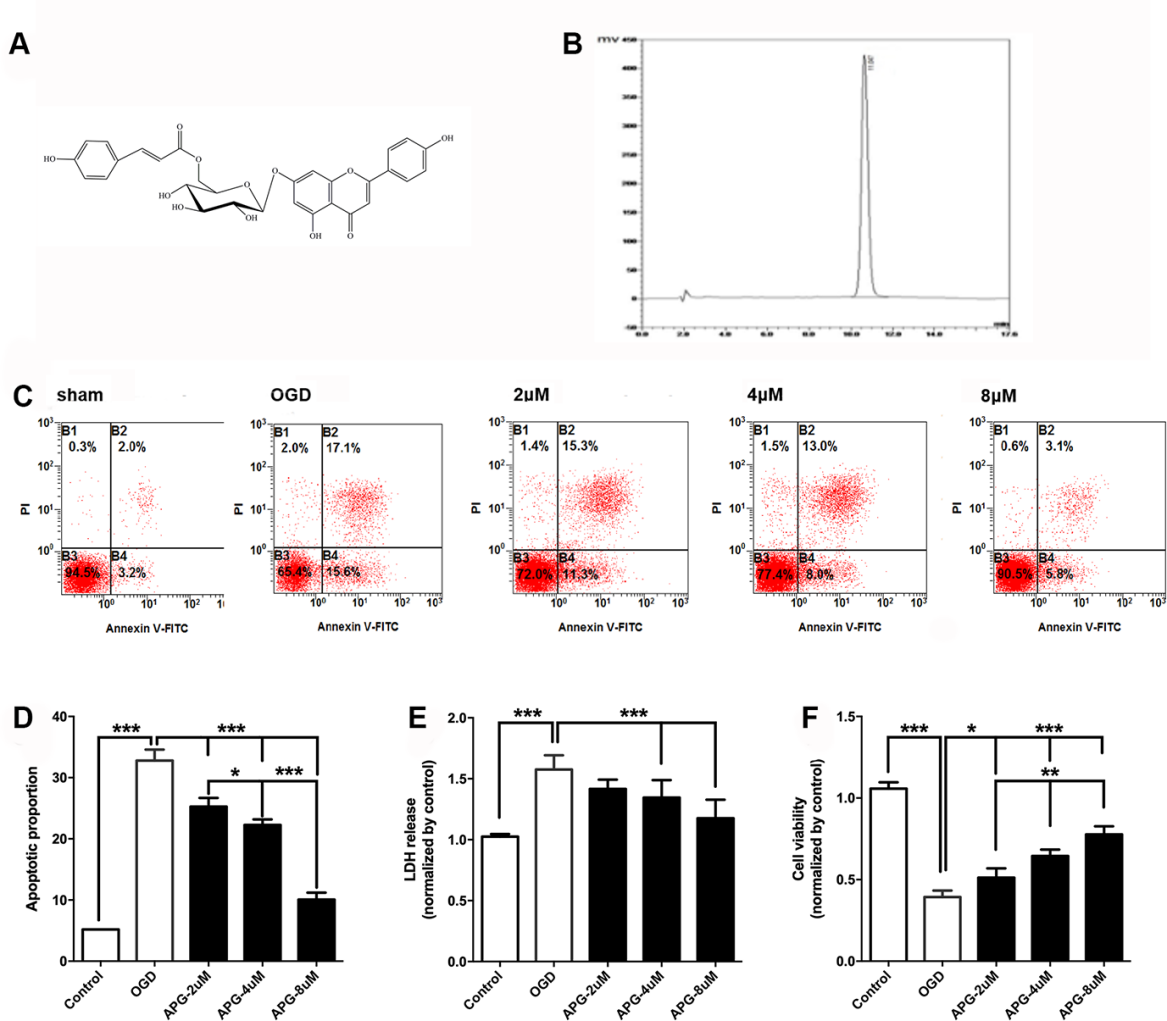
**Protective effect of APG against OGD/R injury in cortical neurons.** (**A**) Chemical structure of APG (molecular weight: 578; molecular formula: C30H26O12). (**B**) HPLC analysis of APG (retention time = 11.047 min). The detection wavelength was 254 nm; the mobile phase was methanol and 0.1% phosphoric acid at a ratio of 40:60 (v/v) through the elution. The flow rate was kept at 1 mL/min. (**C**) Representative dot plots showing flow cytometric analysis of cortical neurons treated with different concentrations of APG and then stained with FITC-conjugated Annexin V and propidium iodide in control, OGD, 2 μM, 4 μM and 8 μM APG treatment groups. (**D**) Analysis result of apoptotic index in each group (n = 5). (**E**) Effect of APG treatment on plasma lactate dehydrogenase (LDH) releasing level in primary cortical neuron culture challenged by OGD/R. (**F**) Effect of APG treatment on cell viability in primary cortical neuron culture subjected to OGD/R. n = 5 per group. * *P* < 0.05, ** *P* < 0.01, *** *P* < 0.001, respectively.

Figure 1A illustrates the chemical structure of APG. The purified APG (purity > 98%) used in the current study was extracted from *C. tangutica,* and the molecular formula was established as C_30_H_26_O_12_ (molecular weight: 578), as reported previously [35]. The chemical structure of APG was identified on the basis of chemical and spectroscopic evidence, and the purity of APG was analyzed using a Shimadzu LC-20A high-performance liquid chromatography system (Figure 1B). Comprehensive flow cytometric analysis results are presented in Figure 1C. The proportion of apoptotic cells in the OGD group was significantly increased in comparison with that in the control group (Figure 1D, *P* < 0.001). APG supplementation (2 μM, 4 μM and 8 μM) prevented the cell apoptosis produced by OGD/R (*P* < 0.001). Moreover, 8 μM APG treatment elicited a more effective protective effect against OGD-induced apoptosis than 2 μM and 4 μM APG (*P* < 0.001).

The level of LDH release in the OGD group was significantly higher than that in the control group (*P* < 0.001), while 4 μM and 8 μM but not 2 μM APG treatment reduced the elevated LDH release compared with that in the OGD group (Figure 1E, *P* < 0.001).

As shown in Figure 1F, cell viability was reduced in the OGD group compared with that in the control group (*P* < 0.001). APG at all three concentrations improved cell viability (2 μM, *P* < 0.05; 4 μM and 8 μM, *P* < 0.001). A dose-dependent protective effect was detected among the treatment groups (2 μM *vs.* 4 μM, *P* < 0.01; 4 μM *vs.* 8 μM, *P* < 0.01).

### APG treatment promoted GSK-3β phosphorylation and Nrf2 nuclear translocation *in vitro*

**Figure 2:**
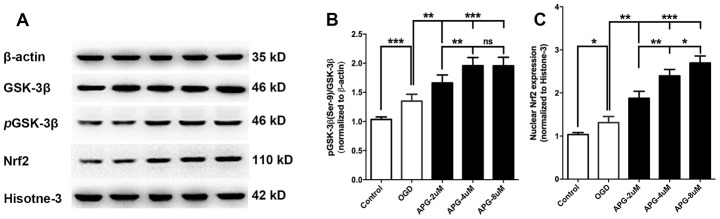
**Effect of APG treatment on phosphorylation of GSK-3β at Ser-9 and nuclear Nrf2 expression.** (**A**) Comprehensive photograph of *p*-GSK-3β (Ser-9), total GSK-3β, and nuclear Nrf2 expression and the corresponding β-actin and histone-3 bands. (**B**) APG treatment increased the phosphorylation of GSK-3β compared with the OGD group. (**C**) Statistical analysis of western blotting result for Nrf2 in the control, OGD, and APG (2 μM, 4 μM and 8 μM) treatment groups. APG treatment increased Nrf2 nuclear expression compared with the OGD group. n = 5 per group. * *P* < 0.05, ** P < 0.01, *** P < 0.001. NS = not significant.

All comprehensive western immunoblotting bands are shown in Figure 2A. As shown in Figure 2B, the phosphorylation level of GSK-3β at Ser9 was increased in the OGD group compared with that in the control group (*P* < 0.001). At all three concentrations, APG improved the phosphorylation of GSK-3β compared to the OGD group (2 μM, *P* < 0.05; 4 μM and 8 μM, *P* < 0.001). Supplementation with 4 μM APG led to higher GSK-3β phosphorylation (*P* < 0.01), and a significant difference was not detected between the 4 μM and 8 μM groups.

Statistical analysis of the Nrf2 nuclear translocation results is presented in Figure 2C. The OGD operation increased Nrf2 expression in the nucleus compared with the control group (*P* < 0.05). APG treatment at 2 μM, 4 μM and 8 μM increased Nrf2 nuclear translocation in comparison with the OGD group (2 μM, *P* < 0.01; 4 μM and 8 μM, *P* < 0.001). A dose-dependent effect of APG treatment on Nrf2 nuclear translocation was detected (2 μM *vs.* 4 μM, *P* < 0.01; 4 μM *vs.* 8 μM, *P* < 0.05). Based on this, 4 μM APG was used in treatment procedures in the following *in vitro* experiments.

### Protective effect of APG treatment was reversed by knockdown of Nrf2 *in vitro*

**Figure 3:**
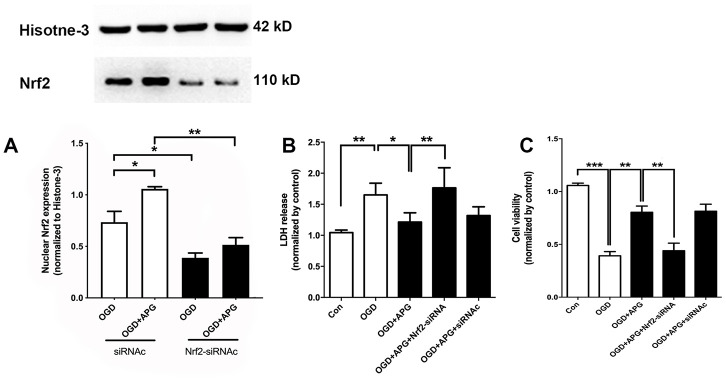
**Knockdown of Nrf2 via RNA interference prevented the nuclear Nrf2 translocation and protective effect induced by APG.** (**A**) The upper portion shows a comprehensive photograph of Nrf2 and the corresponding histone-3 bands. The lower portion shows Nrf2 expression in the presence of Nrf2-siRNA or Nrf2-siRNAc. (**B**) Effect of Nrf2-siRNA or Nrf2-siRNAc on plasma lactate dehydrogenase (LDH) release level in primary cortical neuron cultures treated with APG. (**C**) Effect of Nrf2 knockdown on cell viability in primary cortical neuron cultures with APG treatment. n = 5 per group. * *P* < 0.05, ** P < 0.01, *** P < 0.001.

To further verify the role of Nrf2 in the protective effect of APG treatment, Nrf2 siRNA was applied in this experiment. As shown in Figure 3A, supplementation with Nrf2-siRNA significantly decreased Nrf2 nuclear expression in both the OGD and 4 μM-APG groups compared with the Nrf2-siRNAc-treated groups. As shown in Figure 3B, APG treatment reduced LDH release (*P* < 0.05), while knockdown of Nrf2 prevented the attenuation in LDH expression produced by APG treatment (*P* < 0.01). No significant difference was detected between the APG and APG+Nrf2-siRNAc treatment groups. As shown in Figure 3C, supplementation with 4 μM APG ameliorated the reduced cell viability induced by OGD (*P* < 0.01). However, this improvement was reversed by Nrf2-siRNA (*P* < 0.01), while Nrf2-siRNAc treatment did not alter the protective effect of APG on cell viability.

### PI3K inhibitors reduced the GSK-3β phosphorylation and reversed the increase in Nrf2 nuclear expression induced by APG *in vitro*

**Figure 4:**
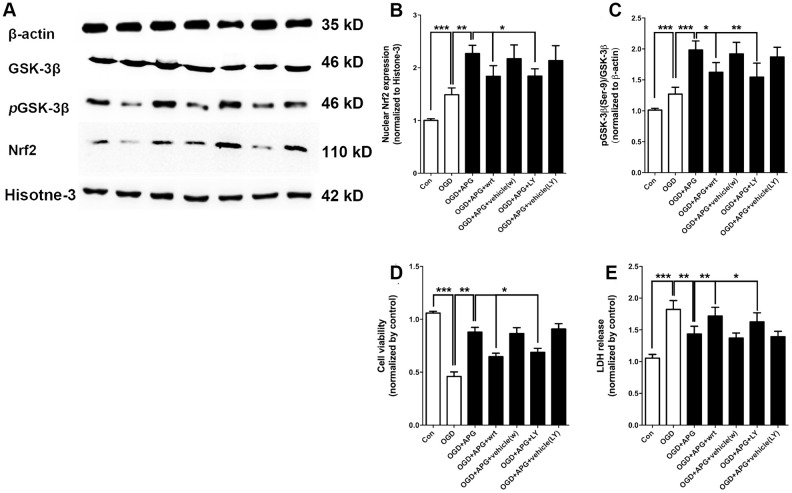
**PI3K inhibitors wortmannin and LY294002 reversed the increase in Nrf2 nuclear translocation and GSK-3β phosphorylation and prevented the protective effect induced by APG.** (**A**) Comprehensive photograph of *p*-GSK-3β (Ser-9), total GSK-3β, and nuclear Nrf2 expression and the corresponding β-actin and histone-3 bands. (**B**) Effect of wortmannin and LY294002 on the increased Nrf2 nuclear translocation mediated by APG. (**C**) Effect of wortmannin and LY294002 on the increased phosphorylation of GSK-3β induced by APG. (**D**) Effect of wortmannin and LY294002 on the plasma lactate dehydrogenase (LDH) release level in primary cortical neuron cultures treated with APG. (**E**) Effect of wortmannin and LY294002 on cell viability in primary cortical neuron cultures with APG treatment. n = 5 per group. * *P* < 0.05, ** P < 0.01, *** P < 0.001.

To further determine the involvement of GSK-3β-induced Nrf2 activation in APG treatment, dephosphorylation of GSK-3β was conducted using the PI3K inhibitors wortmannin and LY294002. Using western immunoblotting analysis (Figure 4A), the phosphorylation of GSK-3β was found to be upregulated in the APG+OGD group compared with the OGD group (*P* < 0.001), while this increased *p*-GSK-3β(Ser-9) level was prevented in both the APG+wrt group and APG+LY group (Figure 4B, *P* < 0.05). As expected, treatment with the vehicle used for wortmannin and LY294002 did not elicit any significant differences with the APG + OGD group. As shown in Figure 4C, the increased Nrf2 nuclear expression produced by APG treatment was reversed in both the APG+wrt group and APG+LY group (APG *vs.* APG+wrt, *P* < 0.05; APG *vs.* APG+LY, *P* < 0.05; respectively). Neither APG+vehicle (w) or APG+vehicle (LY) affected Nrf2 nuclear translocation in comparison with the APG group.

### PI3K inhibitors abolished the protective effect of APG *in vitro*

The improvement in cell viability produced by APG was abolished by wortmannin and LY294002 as compared with the APG+OGD group (Figure 4D, APG vs. APG+wrt, *P* < 0.01; APG vs. APG+LY, *P* < 0.05; respectively). Additionally, both wortmannin and LY294002 reversed any reduction in LDH release seen under conditions of APG treatment as compared with the APG+OGD group (Figure 4E, APG vs. APG+wrt, *P* < 0.01; APG *vs*. APG+LY, *P* < 0.05; respectively).

### APG treatment attenuated cerebral ischemic/reperfusion injury

**Figure 5:**
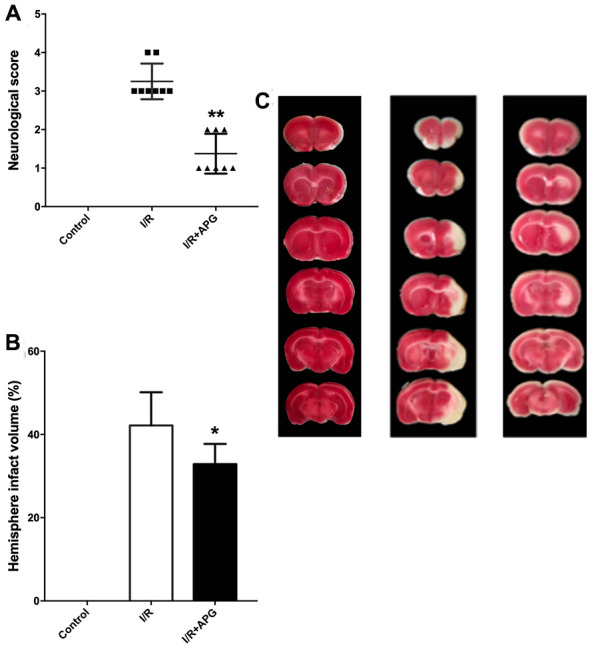
**Neuroprotective effect of APG treatment against focal cerebral ischemic/reperfusion injury in rats.** (**A**) Neurological scores; (**B**) infarct volumes at 72 hours after reperfusion; (**C**) representative photographs of TTC staining. APG treatment significantly ameliorated the neurological scores and reduced infarct size compared with the I/R group (n = 8). * *P* < 0.05, ** P < 0.01. I/R = ischemia/reperfusion.

The neurological score assessment is presented in Figure 5A. A significant neurological deficit score was found in the I/R group compared with the control group. Treatment with APG at 50 mg/kg significantly ameliorated the neurological behavior compared with the I/R group (1.38 [1, 2] *vs.* 3.25 [3, 4], *P* < 0.01). Compared with the I/R group, the 50-mg/kg-APG-treatment group showed a significant reduction in cerebral infarct size (32.88% *vs.* 42.13%, *P* < 0.05).

### APG treatment promoted GSK-3β phosphorylation and Nrf2 nuclear translocation *in vivo*

**Figure 6:**
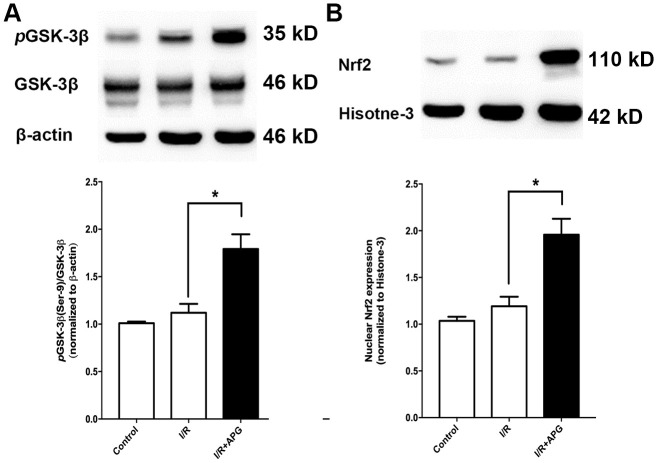
**Effect of APG treatment on *p*-GSK-3β (Ser-9) expression and Nrf2 nuclear translocation.** (**A**). APG treatment increased the phosphorylation of GSK-3β as compared with the I/R group. (**B**) The comprehensive photograph and analysis result of western-blot statistical analysis of Nrf2 in the control, I/R, and APG (50 mg/Kg) treatment groups. APG treatment increased Nrf2 nuclear expression as compared with the I/R group. n = 5 per group. * P < 0.05.

As shown in Figure 6A, GSK-3β phosphorylation in the APG+I/R treatment group was significantly increased compared with that in the I/R group (*P* < 0.05). As shown in Figure 6B, ischemic/reperfusion injury did not significantly affect nuclear translocation of Nrf2, while APG treatment significantly increased Nrf2 protein expression in the nucleus (APG+I/R vs. I/R, *P* < 0.05).

### Both Nrf2 knockdown and PI3K inhibitors reversed the neuroprotective effect and antioxidant effect of APG

**Figure 7:**
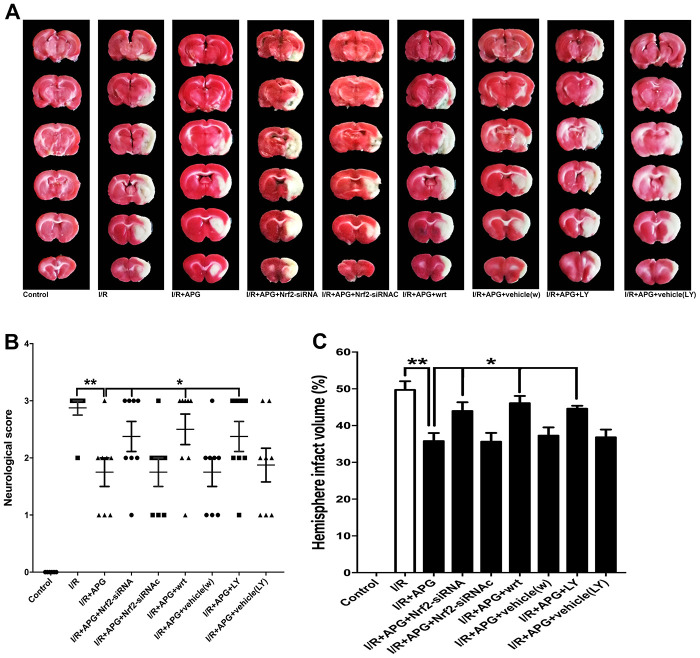
**PI3K inhibitors wortmannin and LY294002 or Nrf2 knockdown reversed the neuroprotection induced by APG treatment.** Representative TTC staining images (**A**), neurological behavior results (**B**), and infarct volume results (**C**) are presented. Both the inhibition of GSK-3β phosphorylation induced by PI3K inhibitors and the Nrf2 knockdown produced by siRNA reversed the neurobiological behavior improvement and increased the infarct volume compared with the APG group (n = 7 per group). * *P* < 0.05 *vs.* I/R+APG group. I/R = ischemia/reperfusion.

As shown in Figure 7 the GSK-3β phosphorylation inhibition produced by wortmannin and LY294002 reversed the neuroprotective effect of APG treatment, as indicated by the worse neurological outcome and larger brain infarct volume than observed in the wild-type mouse group (APG *vs.* APG+wrt; APG *vs.* APG+LY, *P* < 0.05; respectively). Moreover, rats exposed to Nrf2 siRNA also exhibited a worse neurological outcome and larger infarct volume than those in the APG+I/R group (*P* < 0.05).

**Figure 8:**
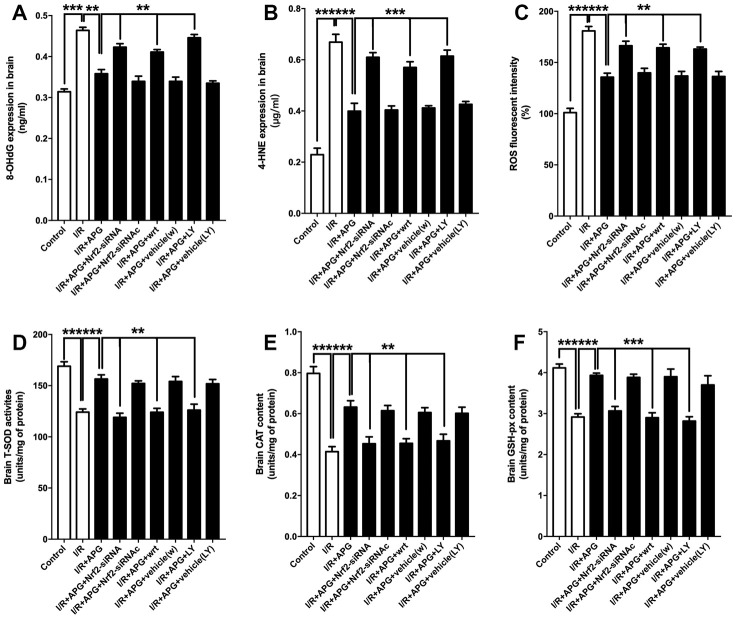
**PI3K inhibitors wortmannin and LY294002 or Nrf2 knockdown abated the increase in antioxidant enzymes and reversed the relief of oxidative stress induced by APG treatment.** Cellular oxidative products and antioxidant enzyme activities are illustrated. Both the GSK-3β phosphorylation inhibition induced by PI3K inhibitors and the Nrf2 knockdown produced by siRNA increased the 8-OHdG (**A**), 4-HNE (**B**) and ROS (**C**) contents and decreased all the content of SOD (**D**), CAT (**E**) and GSH-px (**F**) (n =6 per group). ** *P* < 0.01, *** *P* < 0.001 *vs.* I/R+APG group.

The effect of PI3K inhibitors and Nrf2-siRNA on the level of antioxidant enzymes and oxidative products is presented in Figure 8. As shown in Figure 8A–8C, the GSK-3β phosphorylation inhibition produced by wortmannin and LY294002 reversed any reduction in oxidative product release seen under conditions of APG treatment compared with the APG+I/R group (APG *vs.* APG+wrt; APG *vs.* APG+LY, *P* < 0.01, 0.001; respectively). Moreover, Nrf2 knockdown also reversed the attenuated expression of oxidative products compared with the APG+I/R group (*P* < 0.01, 0.001; respectively). The levels of antioxidant enzymes (Figure 8D–8F; SOD, CAT, and GSH-px) in the APG+wrt, APG+LY and APG+Nrf2-siRNA groups were lower than those in the APG+vehicle(w), APG+vehicle (LY) and APG+Nrf2-siRNAc groups (*P* < 0.01, 0.001).

## DISCUSSION

In this study, the role of GSK-3β phosphorylation-mediated Nrf2 nuclear translocation induced by APG treatment against cerebral I/R injury was studied *in vitro* and *in vivo*. The main findings of this study were as follows: APG treatment improved cell viability and reduced cell apoptosis in primary cortical neurons challenged by OGD/R. Consistent with the *in vitro* results, APG treatment ameliorated neurological outcome and reduced infarct volume in rats subjected to focal cerebral I/R injury. Additionally, this study found that APG treatment upregulated Nrf2 nuclear expression. Knockdown of Nrf2 *via* RNA interference reversed the neuroprotective effect of APG both *in vitro* and *in vivo* and abolished the antioxidant effect of APG in rats. Moreover, APG increased the phosphorylation of GSK-3β, while the dephosphorylation of GSK-3β produced by wortmannin and LY294002 reversed the increase in Nrf2 nuclear translocation and abolished the I/R tolerance induced by APG in cultured neurons and rats. Taken together, these results reveal that GSK-3β-dependent Nrf2 activation plays a crucial role in the neuroprotective effect elicited by APG.

Abundant evidence has demonstrated the vital role of oxidative stress in the pathogenesis of cerebral I/R injury [36]. This suggests that bioactive compounds found to have an antioxidant property might have therapeutic potential by targeting attenuation of oxidative stress in ischemic stroke [37–40]. As a glycoside flavonoid, APG has been demonstrated to have the capacity to scavenge free radicals and augment antioxidant levels [41]. More importantly, based on its liposoluble constituent and small molecular weight, APG is believed to be able to easily cross the blood-brain barrier. Consistent with our previous study, the current study showed that APG elicited a neuroprotective effect, as indicated by the attenuation of OGD/R-induced injury in primary cortical neurons and the induction of tolerance against transient focal cerebral I/R injury in rats.

Our next investigation in this study explored the underlying mechanism of the neuroprotective effect of APG. It has been well demonstrated that several factors, especially Nrf2-regulated AREs, are major contributors to activation of the endogenous antioxidant system [42]. As a member of the NF-E2 family of nuclear basic leucine zipper transcription factors, Nrf2 is a major contributor to the endogenous defense against ischemic oxidative injury [43]. This regulation effect is only produced by accumulation of Nrf2 binding to AREs in the nucleus [44–46]. Therefore, it is of particular interest to demonstrate whether APG elicits its protective effect through Nrf2 nuclear translocation and whether this is in association with activation of endogenous antioxidant enzymes and attenuation of oxidative stress products. In the present study, APG treatment promoted Nrf2 nuclear translocation, which was correlated with a significant increase in expression of the Nrf2-regulated antioxidant genes NQO-1 and GCLC-1 (in Supplementary Figure 1). Transfection of primary cortical neurons with Nrf2 siRNA abolished the protective effect of APG. Moreover, knockdown of Nrf2 via RNA interference in rats reversed the neuroprotective effect and prevented the antioxidant enzyme expression produced by APG. These results indicate that APG exerts its neuroprotective property against oxidative insults via activation of Nrf2.

The first mechanism of Nrf2 regulation identified was inhibition via Keap1-dependent ubiquitination [47]. However, considerable recent advances also demonstrate other regulation mechanisms in determination of Nrf2 activation, including GSK-3β [22, 48]. Thus, we hypothesized that the increased activation of Nrf2 induced by APG may be mediated through GSK-3β. In the current study, the expression of *p*-GSK-3β (Ser-9), which is the inactive form of GSK-3β, was significantly increased in the APG treatment group at 4 h after reperfusion, and these results are consistent with results from other groups. To further elucidate the exact role of GSK-3β in Nrf2 activation induced by APG treatment, two well-established pharmacological strategies of GSK-3β dephosphorylation, wortmannin and LY294002, were employed in this study. The increased nuclear translocation of Nrf2 produced by APG was reversed by supplementation with wortmannin and LY294002. Moreover, consistent with the reduced nuclear translocation of Nrf2, the reduced cell apoptosis after OGD/R in primary cortical neurons and the ameliorated neurological outcome and reduced infarct volume after MCAO in rats were absent in the wortmannin and LY294002 treatment groups.

Some limitations should be noted in the present study. Although this study reported that GSK-3β phosphorylation induced by APG treatment critically increased the nuclear translocation of Nrf2, some recent advances indicate that this signaling pathway increases mitochondrial dynamics [49]. Whether APG regulates Nrf2 activation and increases mitochondrial dynamics requires more investigations in the future.

Based on these *in vitro* and *in vivo* investigations, we conclude that Nrf2 activation downstream of GSK-3β participates in the neuroprotective effect of APG against cerebral ischemia. Overall, this study reveals a new underlying mechanism of ischemic tolerance induced by APG.

## MATERIALS AND METHODS

### Animals

Male Sprague-Dawley (SD) rats aged 8-12 weeks and weighing 250-280 g were obtained from The Experimental Animal Center of the Fourth Military Medical University. All animal-related experimental procedures carried out in this study were approved by the Ethics Committee for Animal Experimentation of the Fourth Military Medical University (Xi’an, Shaanxi, China) and were in compliance with the National Institutes of Health Guide for the Care and Use of Laboratory Animals. Animals were housed under a 12-h alternating light and dark cycle at a room temperature of 20-25° C with 60% humidity. Water and food were freely available except during the 24 h prior to surgery. A randomized number table was used for randomization in the present study.

### Materials

The purified APG (purity > 98%) used in the current study was extracted from *C. tangutica,* and the molecular formula was established as C_30_H_26_O_12_ (molecular weight: 578), as reported previously.

Wortmannin (Sigma Aldrich, USA), a selective phosphatidylinositol 3-kinase (PI3K) inhibitor, was administered intraperitoneally at a dose of 0.6 mg/kg in 10% dimethyl sulfoxide (DMSO) 30 min before middle cerebral artery occlusion (MCAO) surgery. The vehicle group only received the same volume of DMSO. Another selective PI3K inhibitor, LY294002 (Sigma Aldrich, USA), or its vehicle was supplied intracerebroventricularly 10 min before MCAO at a concentration of 15 mM in 50% DMSO in a total volume of 10 μl (coordinates: 0.8 mm anteroposterior, 1.5 mm lateral to the bregma and 3.5 mm deep). The LY294002 vehicle group received an equal volume of 50% DMSO delivered via intraventricular injection.

### Primary cortical neuron culture

Pregnant female Sprague-Dawley rats were euthanized, and cortices from E18 embryos were dissected. After incubation in trypsin-EDTA (0.25%, Gibco) for 10 min, the cortices were dissociated, and neurons were plated in 6-well polylysine-coated plates in DMEM (Gibco)/10% fetal bovine serum (FBS) at a density of 0.6 × 10^6^ cells per well. To identify dendritic morphology, cells were plated on slips with a glass cover at 0.4 × 10^6^ cells per 6-well plate. The medium in both the 6-well plate and coverslips was switched to serum-free neurobasal medium containing B27 (Gibco), which was replaced with fresh medium every 5 days. Cells were maintained at 37° C with 5% CO_2_ and 95% humidity. In determination of neurons, cortical primary neurons were stained with neuron-specific enolase (NSE). All *in vitro* experiments were performed 72 hours after cells were seeded onto plates at an appropriate density according to the procedure for each experiment.

### Oxygen and glucose deprivation/reoxygenation (OGD/R) and APG treatment *in vitro*

OGD/R was applied as an *in vitro* model of hypoxic-ischemic insult. [34] Briefly, the cultured neurons were treated with different concentrations of APG (2 μM, 4 μM, or 8 μM) for 4 h before oxygen and glucose deprivation (OGD). Then, to establish an OGD model *in vitro*, the cells were washed with prewarmed Earle’s balanced salt solution (EBSS) three times and then switched into glucose-free EBSS and exposed to a gas mixture of 95% N2/5% CO2 at 37° C for 2 h. Following OGD, the cultures were transferred to the normoxic incubator and refueled with normal culture medium for reoxygenation for 24 h. Control neuron cultures were placed in EBSS containing 25 mM glucose and incubated under normal culture conditions for the same period.

### Assessment of cell viability and cell damage

Cell viability was measured using 3-(4,5-dimethyl-2-thiazolyl)- 2,5-diphenyl-2-H-tetrazolium bromide (MTT) assays. Briefly, cells were cultured at 15×10^3^ cells per well in 96-well plates. After being washed with phosphate-buffered saline (PBS), the cells were incubated with MTT (5 mg/mL, 20 μL per well) at 37° C for 4 h in accordance with the manufacturer’s instructions. Then, the medium was removed, and 150 μl of DMSO was added to each well. The optical density (OD) at 490 nm was recorded via spectrophotometry with an ELISA Reader (Elx 800, Bio-TEK Instrument, USA). Cell viability is expressed as the percentage changes normalized to the control value.

Cell damage was quantitatively measured via evaluation of the LDH content released from the damaged cells into the culture medium after OGD/R. In brief, cells were treated with 0.5% Triton X-100, and then, the medium containing detached cells was harvested and centrifuged to collect supernatant for examination of LDH activity. LDH release was assessed using an available kit in accordance with the manufacturer’s instructions. The LDH release results are expressed as follows: LDH released (%) = (LDH activity in the medium/total LDH activity) × 100%. Cortical neuron cultures without the OGD/R treatment (control group) represented basal LDH release.

### Flow cytometry for cell apoptosis analysis

Flow cytometry was used to assess cell apoptosis according to our previous report [34]. In brief, primary cortical neurons were washed with 1 × annexin V-FITC binding buffer and then incubated with annexin V-FITC and PI for 15 min at room temperature in a dark box, followed by flow cytometry analysis. The apoptotic cells were counted according to annexin V binding and PI uptake.

### Middle cerebral artery occlusion model (MCAO) establishment and APG treatment in rats

Transient focal cerebral ischemic/reperfusion injury was induced by establishment of an MCAO model as described previously [50]. Briefly, in preparation for surgery, rats were fasted overnight but given free access to water. For the operation, the rats were anesthetized with 10% chloral hydrate (350 mg/kg, *i.p.*). A 3-0 nylon suture with a blunted head was inserted into the internal carotid artery through the right common carotid artery until a middle resistance was felt to obstruct blood flow in the MCA. The suture was maintained in position for 2 h and subsequently removed to allow cerebral blood reperfusion. Throughout the operation, the body temperature of the rats was maintained at 37.0 ± 0.5° C with a heating pad. Moreover, regional cerebral blood flow (rCBF) was monitored with a laser Doppler flowmeter (PeriFlux System 5000; Perimed, Stockholm, Sweden) before, during and after MCAO. Only rats whose cortical rCBF decreased to less than 15% of baseline during ischemia and recovered more than 85% during reperfusion were included for data analysis. At 30 min after reperfusion, 50 mg/kg APG or 10% DMSO (vehicle) was administered via intraperitoneal injection.

### Neurological score and infarct volume assessment

Neurological deficit was assessed at 72 hours after reperfusion by an observer who was blinded to experimental groups according to Longa’s methods [51]. After the neurological outcome evaluation was confirmed, the brains were quickly removed for infarct volume evaluation as previously described. In brief, six brain slices were obtained at 2-mm intervals from the intersection of the lambdoidal suture to the front. The slices were stained with a 2% solution of 2,3,5-triphenyltetrazolium chloride (TTC) at 37° C for 20 min and then fixed with 4% paraformaldehyde for 24 h. Brain slices were photographed (Canon Ixus 220HS, Japan), and the infarct volume was assessed using Adobe Photoshop CS5 (Adobe, US). To correct for swelling, a relative infarct size measure was applied according to the following equation: relative infarct size = (contralateral area - ipsilateral noninfarct area)/contralateral area [52].

### Western immunoblotting assessment

For western blotting of crude *in vitro* cell homogenates, cortical neurons were collected and transferred into RIPA lysis buffer containing 50 mM Tris-HCl (pH = 7.5), 150 mM NaCl, 1% Triton X-100, 0.1% SDS, 1 mM NaVO3, 5 mM NaF and 1 × protease inhibitor cocktail. Cell homogenates were centrifuged at 700 *g,* and the supernatants were collected. Pellets were resuspended and sonicated in RIPA lysis buffer in preparation for the next experimental procedure.

For western blotting of *in vivo* brain tissue, at 4 h post reperfusion, rats were euthanized with an overdose of chloral hydrate, and brains were promptly removed. Ischemic penumbra tissue was dissected according to a previously reported method [53]. Tissue was homogenized in ice-cold RIPA lysis buffer (Beyotime, Nantong, China) containing 1% phenylmethanesulfonylfluoride (PMSF).

For nuclear Nrf2 expression analysis, nuclear protein from cells or brain tissue was extracted using an available extraction kit (Pierce, US) in accordance with the manufacturer’s instructions.

The protein concentration in cell or tissue homogenates was examined using the Bradford method. For western immunoblotting, equal amounts of protein were loaded into each lane of a polyacrylamide-SDS gel followed by electrophoresis. The resolved proteins were transferred to a PVDF membrane, blocked with 5% bovine serum albumin (BSA), and incubated with the appropriate primary antibodies overnight at 4° C. The primary antibodies used are listed as follows: Phospho-GSK-3β (Ser-9) monoclonal antibody, GSK-3β monoclonal antibody (both used at a dilution of 1:1,000; Cell Signaling Technology, US), Nrf2 monoclonal antibody (1:500 dilution; Abcam, US), Histone-3 polyclonal antibody (1:500; Signalway Antibody, US) and β-actin polyclonal antibody (1:1,000 dilution; CWBIO, China). The secondary antibody used was either horseradish peroxidase-conjugated goat anti-rabbit antibody or horseradish peroxidase goat anti-mouse antibody (both diluted at 1:20,000; GSGB-BIO, China). Protein expression or activation changes are presented as the ratio of target protein expression to β-actin (for whole protein) or Histone-3 (for nuclear protein) expression.

### ELISA analysis of antioxidant enzymes and oxidative products

Penumbra samples from the ischemic hemisphere were dissected and homogenized in ice-cold saline according to a 1:10 weight-to volume value. Then, samples were centrifuged at 3000 rpm for 15 min at 4° C, and the supernatant was harvested and stored at –80° C for later ELISA assessment. The protein concentration was determined using the BCA method mentioned above. The antioxidant enzyme (SOD, CAT and GSH-px) and oxidation product (MDA, 8-OHdG and ROS) contents were measured with ELISA assay kits in strict accordance with the manufacturer’s instructions.

### Nrf2-siRNA supplementation *in vitro* and *in vivo*

Knockdown of Nrf2 was achieved with Nrf2 siRNA from Genechem Co., Ltd. (Shanghai, China). Transfection of neurons with siRNA was carried out in accordance with *in vitro* procedures described previously [36]. Transfection of rat brain cells with siRNA was conducted in accordance with an *in vivo* protocol reported previously. Briefly, rats were anesthetized with 10% chloral hydrate (350 mg/kg) and placed in a stereotaxic frame. Next, a stainless-steel cannula was implanted in the unilateral cerebral ventricle. The stereotaxic coordinates were 1.8 mm posterior and 1.5 mm lateral to the bregma and a depth of 4.0 mm from the surface of the skull. A titer of 4 × 10^9^ IU/mL Nrf2-siRNA or an equal amount of control scramble RNA was inserted into the ipsilateral ventricle through the cannula. The determination of Nrf2 knockdown was performed via western immunoblotting 72 h after injection.

The target sequences of the Nrf2-siRNA were as follows: Target sequence: 5’-AAGAGTATGAGCTGGAAAAAC-3’ Sense strand: 5’-UCCCGUUUGUAGAUGACAA-3’Antisense strand: 5’-UUGUCAUCUACAAACGGGA-3’.

### Statistical analysis

The neurological deficit results are presented as the median and interquartile range and were analyzed using a Kruskal-Wallis test followed by a Mann-Whitney U test with Bonferroni post hoc correction. Other values are expressed as the mean ± SD and were analyzed via one-way analysis of variance (ANOVA), followed by Bonferroni correction for a post hoc *t*-test. Statistical significance was determined at *P* < 0.05.

## Supplementary Material

Supplementary Figure 1
