# Supplementary material for: Apigenin-7-O-β-D-(-6”-p-coumaroyl)-glucopyranoside treatment elicits a neuroprotective effect through GSK-3β phosphorylation-mediated Nrf2 activation
Source: Aging (Albany NY). 2020 Nov 18;12(23):23872–88. doi: 10.18632/aging.104050 (PMC7762462; doi:10.18632/aging.104050)
Supplement: Supplementary Figure 1 [file aging-12-104050-s001.pdf]

SUPPLEMENTARY FIGURE

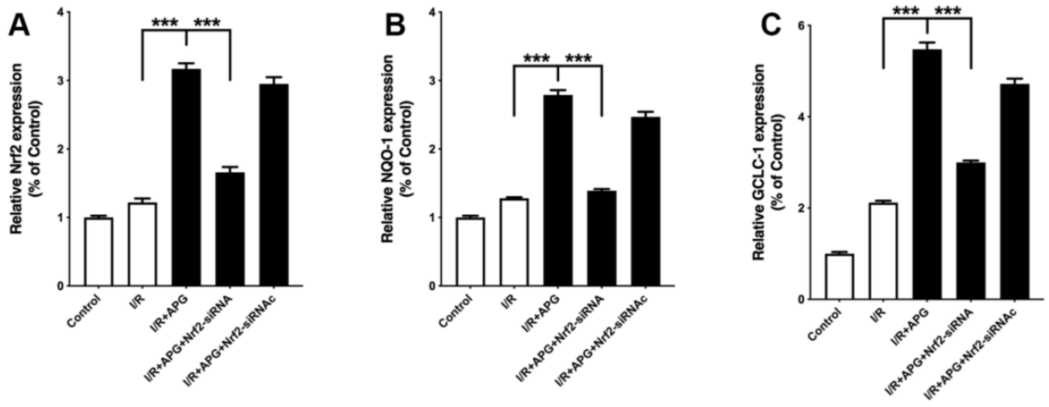

Supplementary Figure 1. APG treatment increased the mRNA expression of Nrf2 and its donstream signal NQO-1 and GCLC-1, respectivelly. (**A**) Nrf2 mRNA expression, (**B**) NQO-1 mRNA expression, (**C**) GCLC-1 mRNA expression at 24 h after reperfusion. *** *P* <0.00.1 I/R = ischemia/reperfusion.
